# DO PUBLIC HOME CARE CLIENTS “LOSE OUT ON SERVICES” BECAUSE THEY HAVE A FAMILY CAREGIVER?

**DOI:** 10.1093/geroni/igad104.0560

**Published:** 2023-12-21

**Authors:** Janice Keefe, Lori Mitchell, Jeff Poss, Jasmine Mah

**Affiliations:** Mount Saint Vincent University, Halifax, Nova Scotia, Canada; Shared Health Manitoba, Winnipeg, Manitoba, Canada; University of Waterloo, Waterloo, Ontario, Canada; Dalhousie University, Halifax, Nova Scotia, Canada

## Abstract

The public home care (PHC) system in Canada is heavily reliant on family caregivers to enable clients to stay in the community. When caregivers are present, there are concerns formal home care aide (HCA) services are less available. Using data from the Resident Assessment Instrument for Home Care (RAI-HC), we examined caregiver and client characteristics in relation to HCA service amounts. Our cohorts were individuals aged 60+ receiving an initial RAI-HC assessment in 2011-2013, and one subsequent assessment in two provincial jurisdictions: the Winnipeg Regional Health Authority (n=5,251) and Nova Scotia Health (n=5291). We measured weekly HCA hours and constructed a predictive model for hours of care controlling for client need. To identify the most underserved client population, we calculated the ratio between predicted to actual received hours of care ascertaining the lowest client quintile. In both jurisdictions, the odds of being underserviced were higher when the client co-resided or had a spouse. Clients living alone at initial intake were least likely to be underserved. Caregiver characteristics increasing the risk of being underserved in Winnipeg included providing IADLs/ADLs support, caring more than 14 hours per week and being unable to continue. Rurality increased risk of underservice in Nova Scotia. These findings contribute to the debate: is PHC a supplement or substitution for family care? PHC as a supplement to family care was more pronounced in Winnipeg and in rural Nova Scotia. Implications for greater equity across regions is explored using an Equity, Diversity and Inclusion lens.

